# Removal of the Greater Portion of Both Upper Jaw-Bones without External Incision

**Published:** 1884-01

**Authors:** 


### Removal of the Greater Portion of Both Upper Jaw-Bones Without External Incision.

The operation was performed by Mr. Bellamy, who thus describes it in the *Medical Times and Gazette*, October 20th, 1883:

Complete anæsthesia being produced, Mr. Bellamy extracted the teeth of the upper jaw with forceps; he then performed Rouge's operation of detaching and raising the upper lip and nose from the superior maxillary bones, and so getting a good view of the anterior chamber. He next passed a small stout saw into the nostril, and divided the hard palate. This was completed by nipping through it with a pair of powerful Liston's forceps. The soft parts were next dissected up from the bones. The removal of the rest of the diseased bone was effected by grasping with lion forceps each lateral half thus divided, wrenching them aside, and cutting away with the Liston forceps all the tissues which appeared to be diseased. Both superior maxillæ as far as the orbital plates were thus removed, and the parts trimmed with strong scissors afterwards; the actual cautery being freely applied to all bleeding points. There was little or no hemorrhage to speak of, and the patient rallied very soon from the operation.

The operation was performed for carcinoma of the entire palate and both superior maxillæ.

